# Melting simulations of high-entropy carbonitrides by deep learning potentials

**DOI:** 10.1038/s41598-024-78377-4

**Published:** 2024-11-19

**Authors:** Viktor S. Baidyshev, Christian Tantardini, Alexander G. Kvashnin

**Affiliations:** 1 Project Center for Energy Transition and ESG, Skolkovo Institute of Science and Technology, Bolshoi Blv. 30, Building 1, Moscow, 121205 Russian Federation; 2 Hylleraas Center, Department of Chemistry, UiT The Arctic University of Norway, PO Box 6050, Langnes, Tromsö, 9037 Norway; 3 Department of Materials Science and NanoEngineering, Rice University, Houston, TX 77005 USA; 4 Institute of Solid State Chemistry and Mechanochemistry SB RAS, Novosibirsk, 630128 Russian Federation

**Keywords:** Condensed-matter physics, Phase transitions and critical phenomena, Structure of solids and liquids, Computational methods

## Abstract

The melting temperature is a crucial property of materials that determines their potential applications in different industrial fields. In this study, we used a deep neural network potential to describe the structure of high-entropy (TiZrTaHfNb)C_x_N_1−x_ carbonitrides (HECN) in both solid and liquid states. This approach allows us to predict heating and cooling temperatures depending on the nitrogen content to determine the melting temperature and analyze structure changes from atomistic point of view. A steady increase in nitrogen content leads to increasing melting temperature, with a maximum approaching for 25% of nitrogen in the HECN. A careful analysis of pair correlations, together with calculations of entropy in the considered liquid phases of HECNs allows us to explain the origin of the nonlinear enhancement of the melting temperature with increasing nitrogen content. The maximum melting temperature of 3580 ± 30 K belongs to (TiZrTaHfNb)C_0.75_N_0.25_ composition. The improved melting behavior of high-entropy compounds by the addition of nitrogen provides a promising way towards modification of thermal properties of functional and constructional materials.

## Introduction

Refractory materials play a key role in various industries where materials should work at extreme conditions, namely extreme heat, thermal shock, and chemical corrosion, making them essential for maintaining the integrity of industrial processes. The widely used materials among ceramics and oxygen- and carbon-based ceramics like magnesium oxide^1^, silicon carbide^2,3^.

When the refractory metals are considered to be those metals melting at temperatures above 1850 °C, twelve metals are in this group: W (melting point 3410 °C^4^), Re, Os, Ta, Mo, Ir, Nb, Ru, Hf, Rh, V, Cr. The combination of these metals together with carbon allows one to obtain high-temperature carbide ceramics and even more intriguing materials as high-entropy ceramics. High-entropy carbide (HEC) ceramics are defined as a solid solution of five or more cation or anion sublattices with high configurational entropy^5^. Nowadays there are many studies devoted to high entropy carbides^6–11^. Addition of another element as nitrogen potentially will increase the high-temperature stability of entire ceramic. The introduction of nitrogen into the lattice of a carbide promotes physical, mechanical, and chemical properties due to the formation of strong hybrid bonds between the d-electrons of the metal component and the s, p-electrons of C/N and the C ≡ N triple bond^12,13^.

Thus, a new class of carbonitrides of transition metals is promising for refractory applications. According to experimental measurements they possess high hardness, oxidation resistance and refractoriness allowing these materials to be used in the production of cutting tools^14–16^. A study of binary carbonitrides proved an improvement in the physical and chemical properties relative to the corresponding mono-compounds^17^. Thus, the idea of increasing the elemental composition of compounds using the high-entropy concept to achieve outstanding properties has the potential^18,19^.

As the experimental measurement of melting temperature is resource-consuming it is much more preferable to use computational approach in order to predict this parameter. Moreover efficient and accurate method for predicting the melting temperature will allow the screening of refractory materials across existing databases. Computational methods for predicting the melting temperature have a long history^20^, which is based on a variety of different computational approaches having different accuracy. Density functional theory (DFT) is actively used to predict melting temperatures of various materials^21,22^. There are also several mechanical methods that can be used to determine the melting temperature. A vibrational-based model of melting was introduced by Lindemann^23^ which explains the melting phenomenon in terms of instability caused by and the average amplitude of thermal vibrations of atoms. Another approach proposed by Born^24^ is based on determination of melting as mechanical instability of the crystal, i.e. if the condition of *C*_44_ > 0 is violated than crystal begins to melt. Despite high accuracy of DFT in predicting the Gibbs free energies of solid and liquid states, and mechanical approaches, thest methods cannot be used directly to describe the formation of homogeneous melt nucleation inside the crystal during the melting. Melting process in terms of atomistic point of view can be successfully simulated and analyzed using the following methods among many others.

*Hysteresis method* is a simulation process that involves two main steps: (1) the system is heated gradually from an initial temperature T_1_ to a final temperature T_2_ at a constant pressure to obtain T^+^ point describing the superheating point; (2) the temperature is gradually decreased from T_2_ to T_1_ and the T^−^ temperature (supercooling) is determined (the temperatures T^+^ and T^−^ could be determined where there is a discontinuity in the volume of the system); (3) the estimated melting temperature could be evaluated using the following proportion $\:{T}_{M}={T}_{+}+{T}_{-}-\sqrt{{T}_{+}{T}_{-}}$^25–27^.

*Two-phase method* is the method involves the exchange of energy between the solid and liquid phases under constant pressure conditions to reach a stable state at the melting temperature^28–31^. As it follows form the name of the method, the determination of the melting temperature comes from the simulation of coexistence between the solid and liquid phases of a particular compound. This technique is the most accurate for multicomponent compounds^32^. The main limitation of this method is the need to consider relatively large supercells in order to achieve a stable solid-liquid interface. Thus, the prediction of melting temperatures is quite complicated and resource consuming in terms of ab initio simulations but is possible with molecular dynamics simulations with empirical or machine learning potentials.

Recent advancements in machine learning interatomic potentials (MLIPs) open new prospective ways for accurate predictions. Unlike empirical potentials, MLIPs employ various representations of crystal structures, including methods such as GAP^33^, MTP^34^, NNP^35,36^, DeepMD^37^, etc. The application of machine learning (ML) techniques in atomistic simulation of materials has gained significant momentum in the last decades^38–42^.

Here we developed the DeepMD potentials to study the melting temperature of refractory TiZrTaHfNbC_x_N_1-x_ carbonitride ceramics. Potential was trained on the ab initio molecular dynamics trajectories allowing to achieve high accuracy for our predictions. Our approach aims to extend the capabilities of classical molecular dynamics simulations, enabling accurate simulation and analysis of the melting process with prediction of the melting point of high-entropy carbonitrides. Dependence of the melting temperature on the nitrogen content in the considered HECNs was determined and analyzed.

## Computational details

### Training of machine learning potential

**Fig. 1:**
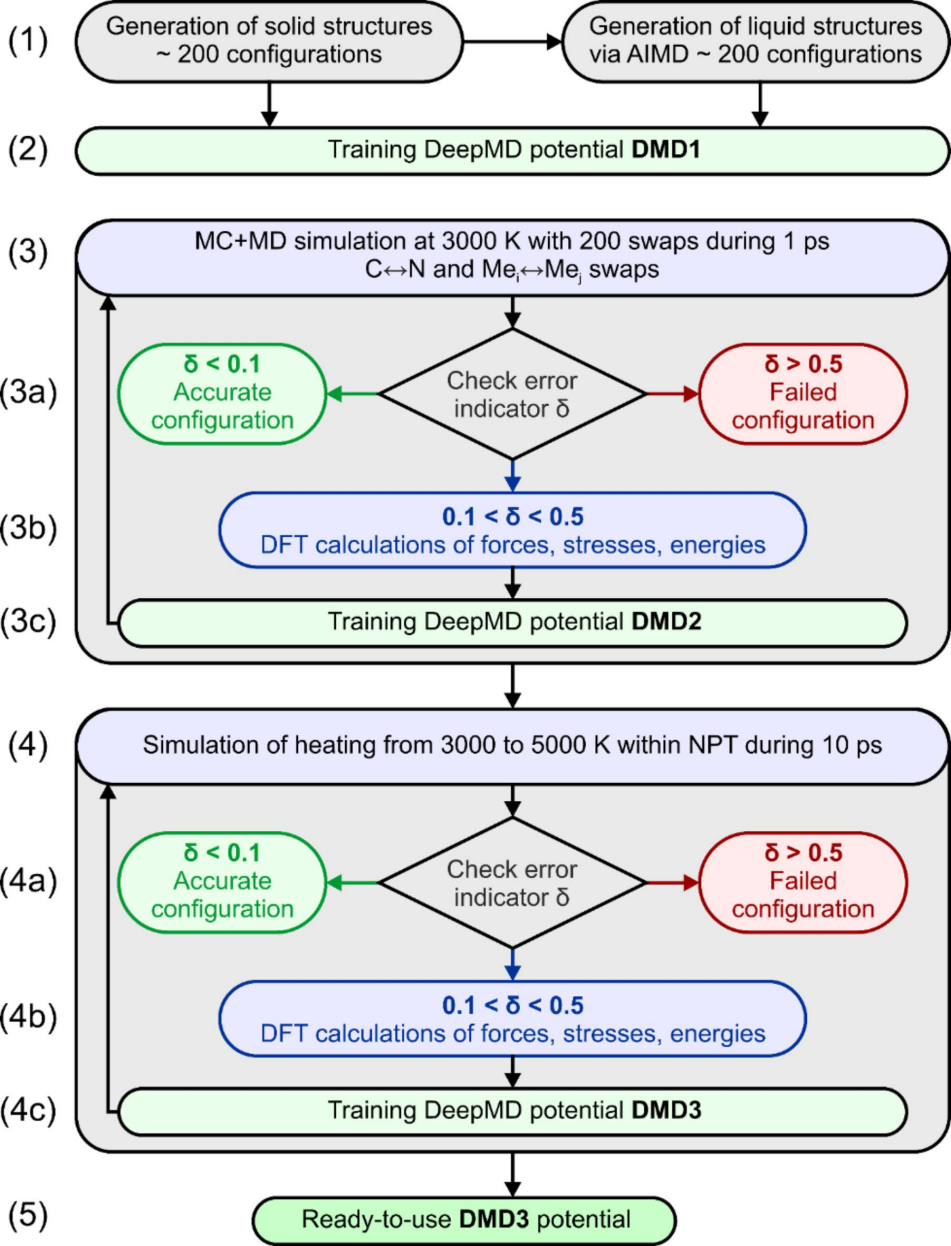
Principal scheme of training the potentials for prediction of melting temperature.

Training of deep neural network (DNN) potential was performed by using the DP-GEN package^43^, which was previously successfully used for training of DNN potentials and simulations^44^. Here the interactive or active learning process is used. The learning process consists of iterations, each iteration is divided into three stages: training, exploration, and labelling. In the first iteration, small datasets are generated from ab initio molecular dynamics (AIMD) calculations (see (1) in the Fig. 1). In this step, we considered HECN structure in the crystalline and liquid states, and for each structure we performed AIMD simulations at a constant temperature of 3500 K during the 200 steps with a timestep of 1.5 fs. AIMD simulations were performed in the VASP code^45–47^. This training set was used to obtain pre-trained DMD1 potential (see (2) in the Fig. 1).

At the next stage (exploration) we used the LAMMPS package^48^ to perform classical molecular dynamics simulations with the pre-trained potential and select candidates for further training. We used two MD simulations that have followed in succession one after the other.

In the first MD simulation, we simultaneously used Monte Carlo and molecular dynamics simulations at a temperature of 3000 K where metal atoms were randomly swapped between each other, and C and N atoms were also swapped during the simulation (step (3) in the Fig. 1). MD was carried out within the NVT ensemble with a time step of 0.5 fs with a total simulation time of 1 ps. During this time the system was allowed for 200 swaps between all types of atoms (200 × 5 = 1000 for metal atoms and 200 swaps between C and N atoms). In the case of HEC and HEN only metal atoms were swapped.

During the simulation we performed the selection of configurations according to the model rejection criterion δ, defined by the maximum forces acting on the atoms^43^ (see (3a) in the Fig. 1). We have chosen a criterion according to which configurations are identified as accurate when δ < 0.1 eV/Å, as failed when δ > 0.5 eV/Å, and as candidates in the intermediate case. From the set of candidate configurations, a small number of configurations are randomly selected for labelling, in our case 150 (30 for each system), (3b) in the Fig. 1. At the labelling stage, the candidate configurations are calculated using DFT methods (see (3b) in the Fig. 1). To obtain the energies and forces acting on the atoms, the only one self-consistent calculations for electronic relaxation is performed without structural relaxation. After about 100 iterations of such learning procedure, we obtained second generation of the potential, namely DMD2, see (3c) in the Fig. 1.

Next, the structures obtained from MC were heated within the NPT ensemble from 3000 to 5000 K for 10 ps (step (4) in the Fig. 1). This allowed one to melt the crystal and obtain the liquid phase. We have considered the following structural compositions as initial for simulations of liquid phases: TiZrTaHfNbC_100_N_0_, TiZrTaHfNbC_75_N_25_, TiZrTaHfNbC_50_N_50_, TiZrTaHfNbC_25_N_75_, TiZrTaHfNbC_0_N_100_. We considered pure HEC, HEN and mixed HECN with C-75%N-25%, C-50%N-50%, C-25%N-75% concentration. Each structure had a size of 9.07 Å×9.07 Å×9.07 Å and contained 64 atoms in total. During the simulation of liquid phase the active learning procedure, (4a)-(4c) in the Fig. 1, similar to steps (3a)-(3c) was applied. After about 100 iterations the final potential DMD3 (step (5) in the Fig. 1) was obtained. DMD3 potential then was used for the rest of simulations.

In total there are about 200 iterations of learning during which about 9000 new configurations were selected, energies, forces, and stresses were calculated and added to the potential DMD3.

For each trained DMD potential the sizes of the embedding and fitting nets are set as (25, 50, 100) and (120, 120, 120), respectively. The cut-off radius is set to 6 Å. Each model is trained with 5000 gradient descent steps with an exponentially decaying learning rate from 10^− 4^ to 10^− 8^.

### Details of DFT calculations

Our calculations are based on the density functional theory (DFT)^49,50^ within the generalized gradient approximation with Perdew–Burke–Ernzerhof functional (PBE)^51^ and two revised versions, namely rPBE^52^ and PBEsol^53^ with the projector augmented wave method^54,55^ as implemented in the VASP^45–47^ code. The plane wave energy cutoff of 400 eV, the Methfessel–Paxton smearing^56^ of electronic occupations, and Гcentered kpoint meshes with a resolution of $\:2\pi\:\times\:0.025$ Å^−1^ for the Brillouin zone sampling were used, ensuring the convergence of the energy differences and stress tensors.

**Fig. 2:**
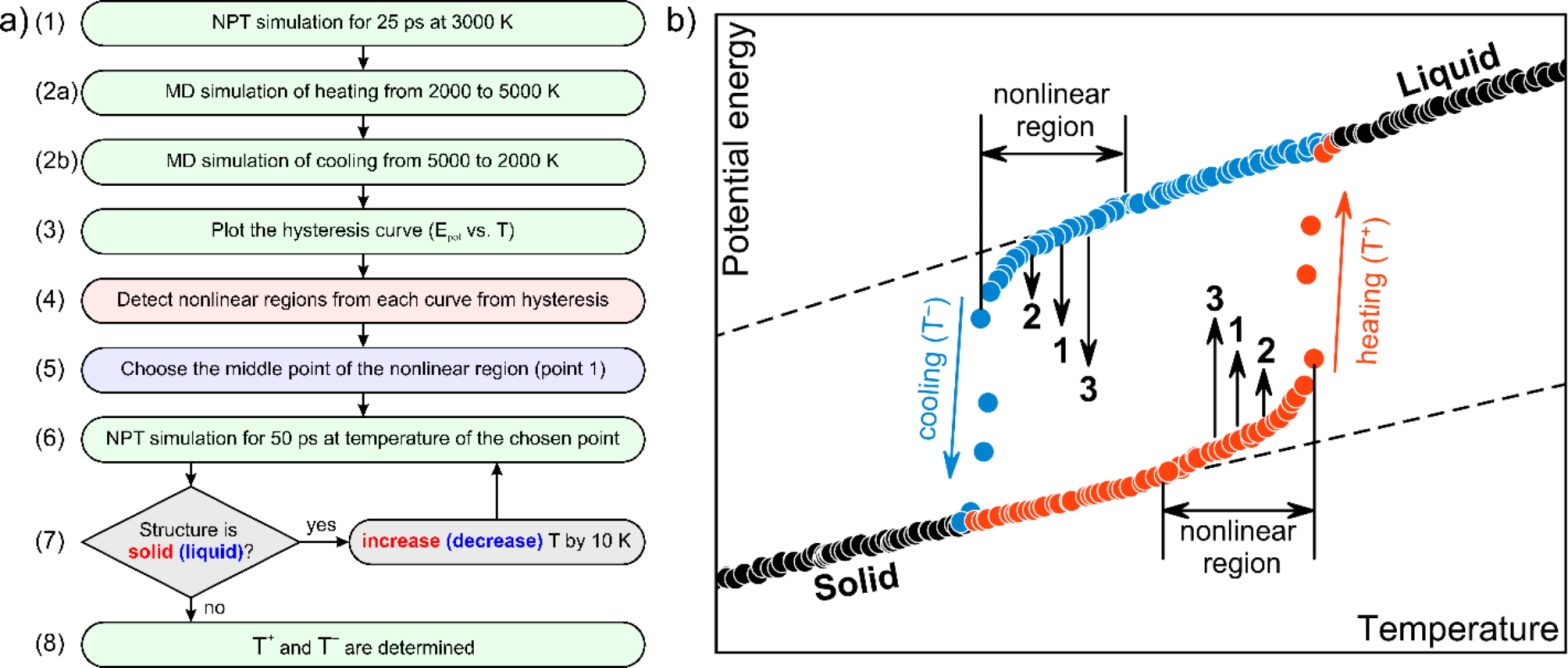
(**a**) Principal scheme of determination of superheating (T^+^) and supercooling (T^–^) temperatures of considered structure. (**b**) Schematic melting hysteresis curve. Heating cycle is shown by red, while cooling is shown by blue color. Solid and liquid regions are shown by black color.

To check which functional, namely PBE, PBEsol, and rPBE, is more suitable for simulations of melting and determination of melting temperature we have performed preliminary training of DeepMD potentials for HfC system. Training procedure presented in Fig. 1 was used for training potential for HfC. During the active learning steps (3a and 4a in Fig. 1) there are 2846, 2395, and 3800 new configurations were sampled for PBE, PBEsol, and rPBE functionals respectively. Using trained potential the procedure described in Fig. 2 is employed to determine superheating (T^+^) and supercooling (T^–^) temperatures of HfC, which are presented in Table 1. The highest difference between prediction and reference experimental data does not exceed 10%. It is known, that PBE functionals may underestimate the melting temperature of carbides compared to experimental data^13,57^. One can see that DMD potential trained on the rPBE data shows the best agreement with reference data on melting temperature for HfC. As a results, all further DMD potentials for simulations are trained with rPBE functional.

**Table 1:** Determined superheating, supercooling and melting temperatures for HfC using PBE, PBEsol, and rPBE functionals. T* is the melting temperatures from reference data.

HfC (4096 atoms)	T^+^, K	T^–^, K	T_M_, K	T*, K
PBE	4441	2962	3776	4232 ± 84^58^ 4100^59^ 3962^13^ 3673^60^
PBEsol	4443	2998	3792	
rPBE	4347	3122	3785	

## Results and discussion

### Accuracy of DMD potential

**Fig. 3:**
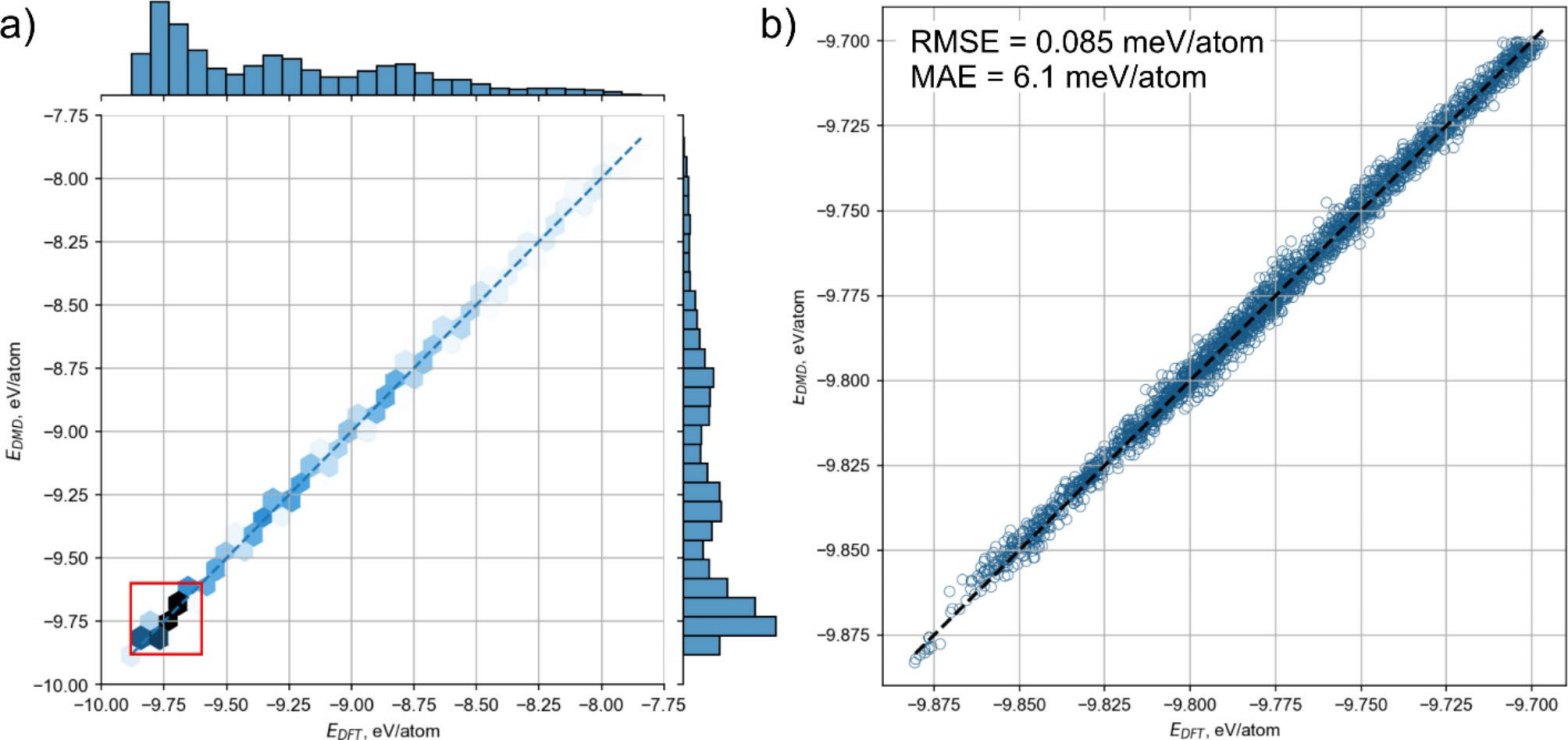
The correlation plots for (**a**) energy per atom. (**b**) Zoomed region highlighted by red contains the highest density of data points.

To be sure about the correctness of obtained results on the melting temperature we should proof the accuracy of trained potentials. The potential, obtained according to developed scheme (DMD3 in Fig. 1) showed high accuracy. The correlation plot for the energy is shown in the Fig. 3a, where the data for 9014 collected configurations are shown. Root mean square (RMS) error in energies per atom predicted by DMD3 potential are about 0.084 meV/atom, with the maximum absolute error of 6.1 meV/atom.

One can note from the Fig. 3a that maximum density of the data concentrated in the regions from − 9.875 to -9.7 eV/atom, which correspond to the energy range of solid configurations. This region is enlarged and shown in Fig. 3b. Configurations which are chosen for training distinguish from each other by the distribution of metal and carbon/nitrogen atoms in the volume of the cell. Small deviation in total energy indicates about the energetic identity of all considered structures. Energy changes with increase temperature when atoms are out of equilibrium. However, the disordered structures do not show greater error in the energy predictions. Thus, we can conclude that DMD3 potential trained within the developed procedure described in Fig. 1 can be used to calculate the melting curves of high-entropy materials.

### Melting simulation

Simulation of melting process of structures with different compositions are carried out within the NPT ensemble. Periodic structures with the 36 × 36 × 36 Å containing 4096 atoms are considered. The melting temperature is determined using multi-step procedure. The principal scheme of this procedure is shown in Fig. 2a.

First, the structure is held at constant temperature of 3000 K for 25 ps, (1) in Fig. 2a, then structure is heated from 3000 to 5000 K during 250 ps (see (2a) in Fig. 2a), which corresponds to a heating rate of 8 × 10^12^ K/s. Then the cooling of the system simulated from 5000 to 3000 K with the same rate as for heating (see (2b) in Fig. 2a). Temperature and potential energy for heating and cooling simulations are averaged over time with a step of 2.5 ps. These steps allow us to obtain a classical heating and cooling curves (step (3)) as shown in the Fig. 2b by red and blue colors.

More attention should be paid to the transition region of each of obtained curves as due to the high heating (cooling) rate we may have skipped the transition region. Next step, (4) in the Fig. 2a, we determine the nonlinear region of the curve close to the transition (Fig. 2b). Then we choose the middle point of the nonlinear region on both curves (see (5) in Fig. 2a) and start the simulation at constant temperature (NPT) corresponding to the velocity distribution at this step during the 50 ps, (6) in Fig. 2a. Subsequently, it is necessary to check whether the structure has undergone a transition to a liquid state (or solid state for the cooling curve), (7) in Fig. 2a. If the structure remains in the solid state (or liquid for cooling) at the end of simulation, the temperature will increase (decrease) by 10 K, and the process will proceed to point 2 in Fig. 2a. Thereafter, the simulation will be repeated from this point for a duration of 50 ps. This procedure is repeated until a liquid phase is obtained for the heating curve or a solid phase is obtained for the cooling curve, (8) in Fig. 2a. The temperatures at which the transition to the liquid (solid) phase occurred are defined as T^+^ and T^–^. Then melting temperature is determined as $\:{T}_{M}={T}_{+}+{T}_{-}-\sqrt{{T}_{+}{T}_{-}}$. For the heating curve, if the structure goes into the liquid phase during the first 50 ps, (6) in Fig. 2a, we reduce the temperature by 10 K and move down the melting curve (point 3 in Fig. 2b). Similar for the cooling curve, if the structure goes into the solid phase, we increase the temperature and move to the point 3 on the cooling curve in Fig. 2b. We repeat this procedure until a solid phase was obtained for heating and liquid phase for cooling to determine T^+^, T^–^ and then T_M_. The solid and liquid phases are determined by changes in the Radial Distribution Function (RDF) using the OVITO package^61^ and by potential energy jump. For each concentration an ensemble of structures consisting of 10 different (mixed) configurations was considered.

### Melting temperature of high-entropy carbonitrides

**Fig. 4:**
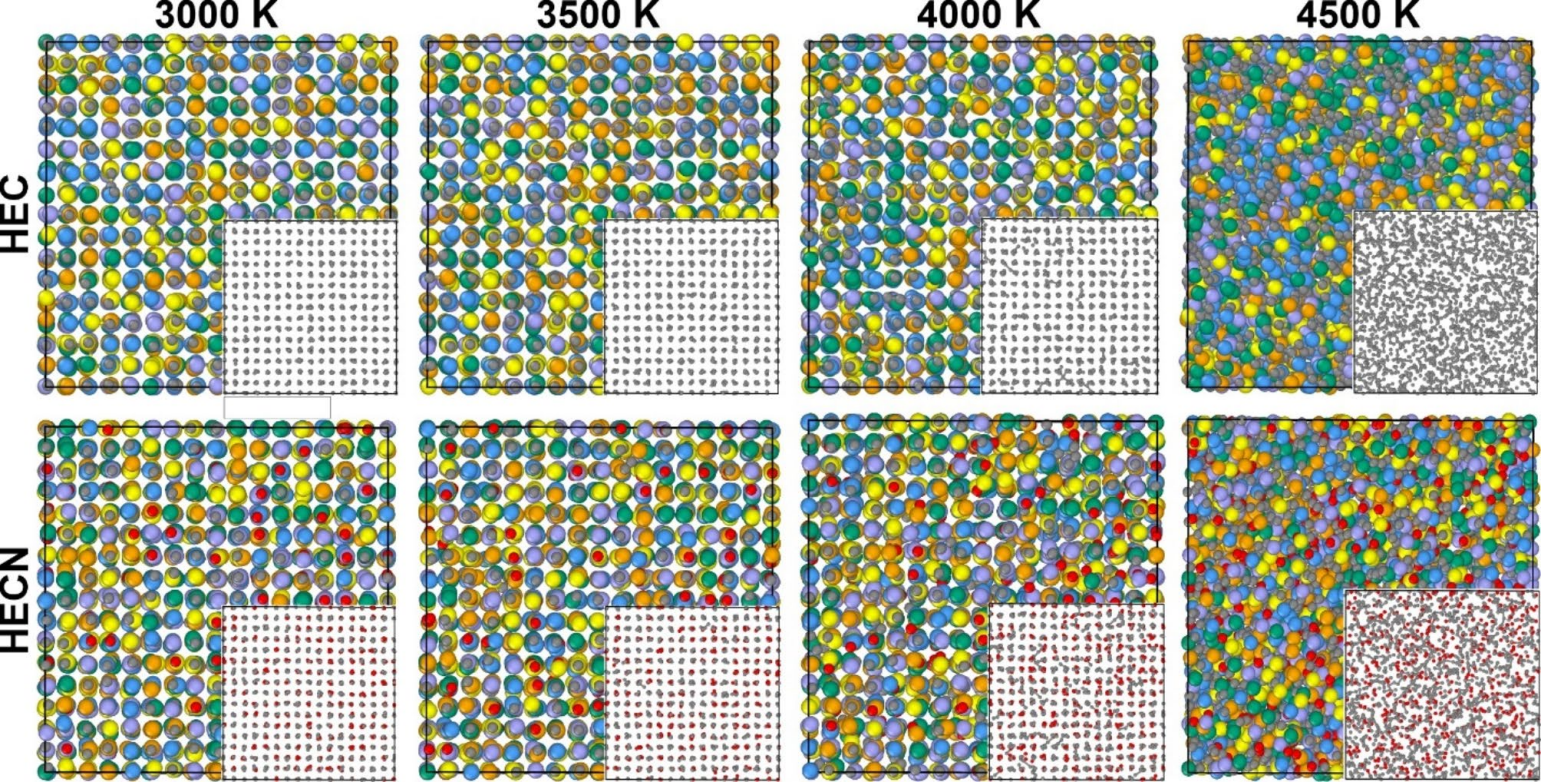
Simulated crystal structures of HEC and HECN (TiZrTaHfNb)C_0.75_N_0.25_ at 3000, 3500, 4000, and 4500 K (liquid) respectively. In the insets the non-metal sublattices are shown. Carbon atoms are shown by gray, while nitrogen is red.

Developed approach is applied for calculations of melting temperature for (TiZrTaHfNb)C_1-x_N_x_ carbonitrides, where *x* changes from 0 to 1 with increment of 0.25. Crystal structures of simulated compounds at 3000, 3500, 4000, and 4500 K during heating are shown in Fig. 4. One can see that in the solid state the non-metal sublattice displays ordered structure with only metal-carbon bonds, while at increased temperature (4500 K) carbon-carbon bonds appears and carbon chains form (detailed description is presented below). For carbonitride we observed similar situation, i.e. the formation of C-C, C-N and N-N bonds, which is analyzed below. The temperature of 4000 K represents a condition close to the melting temperature, where some regions with C-C bonds for HEC and C-C, C-N, N-N bonds for HECN are formed indicating the nucleation of the liquid phase.

**Fig. 5:**
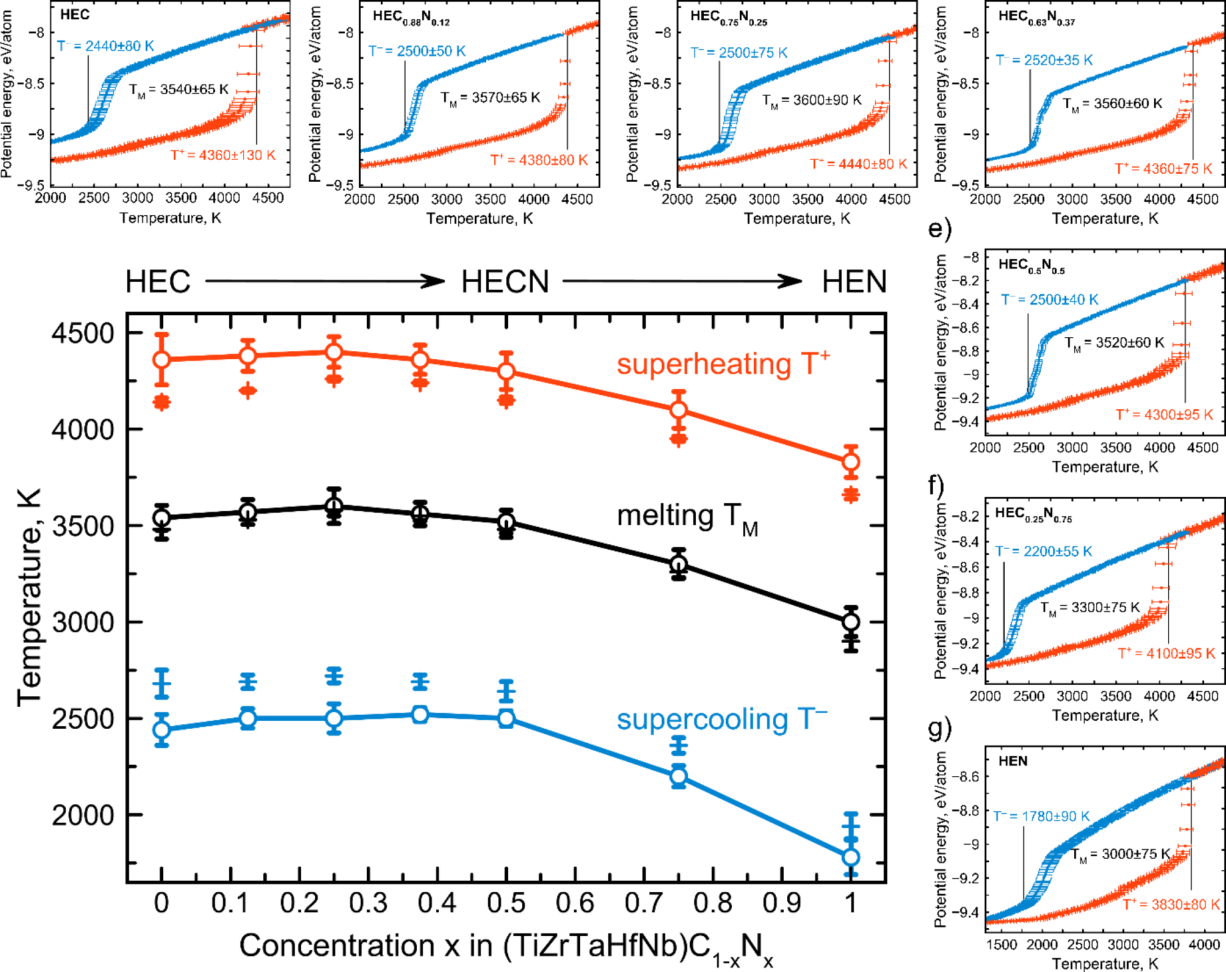
Dependence of the superheating (red) and supercooling (blue) temperatures of (TiZrTaHfNb)C_1-x_N_x_ on the nitrogen concentration *x*. Error bars come from the consideration of 10 configurations for each composition. Open circles indicate data obtained from the curves presented in top and right panels, while crosses show refined temperatures obtained by developed procedure (Fig. 3a). Top and right panels show calculated melting hysteresis with heating (red) and cooling (blue) curves for studied high-entropy carbonitrides, namely HEC, HEC_0.88_N_0.12_, HEC_0.75_N_0.25_, HEC_0.63_N_0.37_, HEC_0.5_N_0.5_, HEC_0.25_N_0.75_, HEN.

Simulated heating (red) and cooling (blue) curves for studied compounds obtained using only steps (1) and (2) from Fig. 2a are shown in top and right panels of Fig. 5. Since we considered 10 different configurations for each composition, there are variations in the simulated solid-liquid and liquid-solid transitions. Considering high-entropy carbide ((TiZrTaHfNb)C, HEC, Fig. 5a) one can see that superheating temperature is determined to be 4360 K, with the error of 130 K associated with different configurations, while supercooling temperature is 2440 ± 80 K (resulting T_M_ = 3540 ± 65 K). Similar situation observed for (TiZrTaHfNb)C_0.75_N_0.25_ (HEC_0.75_N_0.25_ in Fig. 5) structure where the difference between superheating and supercooling temperatures is about 2000 K. The error in determination of temperatures (T^+^, T^–^, T_M_) for other considered carbonitrides is not more than 100 K. One can note the lowest melting temperature to HEN, which is 3000 ± 80 K. Reducing the nitrogen content (HEN◊HEC) leads to an increase in melting temperature. However, it was expected that the highest melting temperature should be devoted to HEC, but our results show the enhanced T_M_ of 3600 K for HEC_0.75_N_0.25_ structure compared to HEC, see Fig. 5.

For each composition we have refined the superheating and supercooling temperatures by applying the rest of steps described in Fig. 2a, namely (3)-(7). To study in more detail the peculiarity in the behavior of melting temperature on the nitrogen concentration we plotted the dependence of refined temperatures as shown in the main panel of Fig. 5. Refined values are shown by crosses, while temperature values obtained directly from the melting curves are shown by open circles. Refined values of superheating, supercooling and melting temperatures together with those obtained from melting curves are summarized in Table 2. As one can see, refining procedure mainly influences on the supercooling temperature, which becomes higher after refining. At the same time the superheating temperature becomes lower after refining. Thus, calculated melting temperatures almost do not change. We have observed nonlinear behavior for temperature dependencies on the nitrogen concentration, see Fig. 5. Addition of small amount of nitrogen leads to slightly increased melting temperature from 3480 ± 50 K for HEC to 3580 ± 30 K for HEC_0.75_N_0.25_. To analyze this region of concentrations we additionally simulated the melting curves and calculated the melting temperatures for two concentrations close to HEC_0.75_N_0.25_ to verify the increase in temperature. Calculated melting temperatures for HEC_0.88_N_0.12_ and HEC_0.63_N_0.37_ are presented in Table 2 and shown in Fig. 5 as well. Based on this information we can conclude that addition of small amounts of nitrogen increases melting temperatures. However, as one can note, further increases in nitrogen content leads to decrease to 2900 ± 50 K for HEN. It should be noted that refined melting temperatures are calculated with less error compared to data from the melting curves, see Fig. 5.

**Table 2:** Calculated superheating, supercooling, and melting temperatures of studied high-entropy compounds from melting curves and refined data.

Compound	From melting curves			Refined		
	T^+^, K	T^–^, K	T_M_, K	T^+^, K	T^–^, K	T_M_, K
HEC	4360 ± 130	2440 ± 80	3540 ± 65	4140 ± 20	2680 ± 70	3480 ± 50
HEC_0.88_N_0.12_	4380 ± 80	2500 ± 50	3570 ± 65	4200 ± 10	2690 ± 35	3530 ± 25
HEC_0.75_N_0.25_	4400 ± 80	2500 ± 75	3600 ± 90	4260 ± 15	2720 ± 35	3580 ± 30
HEC_0.63_N_0.37_	4360 ± 75	2520 ± 35	3560 ± 60	4240 ± 10	2690 ± 35	3550 ± 25
HEC_0.5_N_0.5_	4300 ± 95	2500 ± 40	3520 ± 60	4150 ± 20	2640 ± 50	3480 ± 40
HEC_0.25_N_0.75_	4100 ± 95	2200 ± 55	3300 ± 75	3950 ± 15	2360 ± 40	3260 ± 30
HEN	3830 ± 80	1780 ± 90	3000 ± 75	3660 ± 25	1940 ± 65	2900 ± 50

## Discussion

Considering refractory materials, it is possible to define an effective and well-known approach to achieving a higher melting point, which is related to nonstoichiometric compositions with a depletion of carbon in carbides^62^. The compounds with the highest melting temperature belong to solid solutions of HfC and TaC. The enhanced melting temperature can be achieved by the formation of C-vacancies^62^ or by substituting carbon by nitrogen^13^. It was shown^13^ that the addition of nitrogen significantly changes the liquid structure due to the instability of the C–N and N–N bonds. The liquid is stabilized by its higher entropy, which offsets its higher enthalpy. In particular, the higher entropy of the liquid is reflected by its large variety of pairwise correlations. To illustrate, the entropy of a liquid is greater than that of an ideal gas with the two-body exceeding entropy ($\:{S}_{ex}/{k}_{B}$), being the dominant contributor. This can be defined as follows:

$$\:\frac{{S}_{ex}}{{k}_{B}}=-2\pi\:\rho\:\sum\:_{i,j}{x}_{i}{x}_{j}\underset{0}{\overset{\infty\:}{\int\:}}\left({g}_{ij}\left(r\right)\text{ln}\left({g}_{ij}\left(r\right)\right)-\left({g}_{ij}\left(r\right)-1\right)\right){r}^{2}dr,$$

where *ρ* is the atomic density, *x*_*i*_ and *x*_*j*_ are the fractional compositions of elements in high-entropy compound between which the pair correlations are calculated, *g*_*ij*_ is the pair correlation function between the elements *i* and *j*, and *r* is the distance between atoms *j* and *i.*

**Fig. 6:**
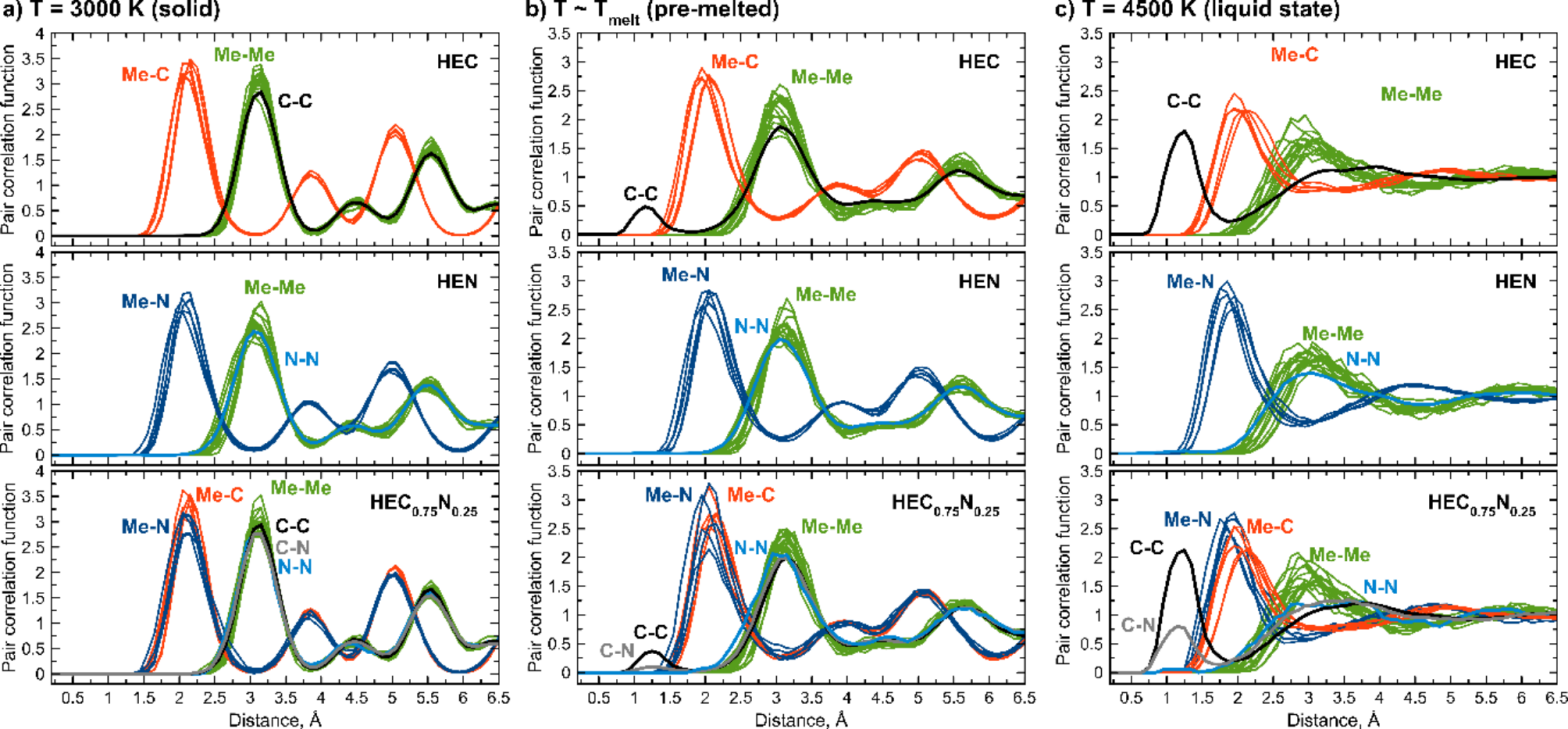
Pair correlation functions (normalized as r → +∞) in (**a**) solid state (T = 3000 K), (**b**) pre-melted state (T ~ T_melt_), and (**c**) liquid-state simulated for HEC, HEN, and HEC_0.75_N_0.25_.

Pair correlation functions are calculated for solid, pre-melted, and liquid phases of HEC, HEN, and HEC_0.75_N_0.25_ obtained as shown in Fig. 6a–c. Structure of (TiZrTaHfNb)C_0.75_N_0.25_ (HEC_0.75_N_0.25_, HECN) is chosen as it possesses highest melting temperature among considered carbonitrides. Considering the solid state of high-entropy materials a similarity between their pair correlation functions can be observed (Fig. 6a). The dominant first neighbor of C and N in HEC, HEN, and HECN is metal (see red and blue Me-C and Me-N correlation peaks). Here we do not distinguish metals by type. Distances between C/N and Me atoms is about 3.15 Å for all compounds. This is due to the small changes in lattice parameters during the transition from HEC to HEN with the substitution of C by N. Pair correlation functions calculated for the pre-melted state are shown in Fig. 6b. One can clearly see the differences between HEC, HECN, and HEN in this state, as part of the volume is now partially in the liquid state. For HEC the dominant first neighbor of C becomes carbon, while the positions of the Me-C correlation peaks do not change. It can be stated that initialization of the melting starts from the formation of stable C-C carbon chains. A similar situation is observed for HECN, where the first correlation peaks are C-C, and C-N (Fig. 6b), indicating the stability of C-C and C-N bonds. For HEN the first correlation peak is Me-N rather than N-N showing significantly different behavior.

The most interesting and important analysis can be done for liquid states of considered compounds. It is evident that the dominant first neighbor of C in HEC liquid is carbon (Fig. 6c). It again evidenced about greater stability of C-C bonds, which forms a strong first neighbor correlation peak, where even C chains with more than three C atoms are found in the simulated atomic structure. Second neighbor peak belongs to Me-C bonds. In contrast, for HEN (Fig. 6c) the first neighbor of N is metallic atom, rather than another nitrogen. This indicates low stability of the N–N bonds in the liquid HEN phase. First correlation peak for Me-Me bonds almost coincides with N-N peak, Fig. 6c, which again differs from HEC. Considering liquid state of HECN one can see additional correlation related to C-N bonds, Fig. 6c. The intensity is lower compared to C-C, which is also indicates about low stability of the C–N and N–N bonds in the liquid HECN phase. These peculiarities in pair correlation functions of HECN compared to HEC and HEN may lead to differences in melting temperature.

**Fig. 7:**
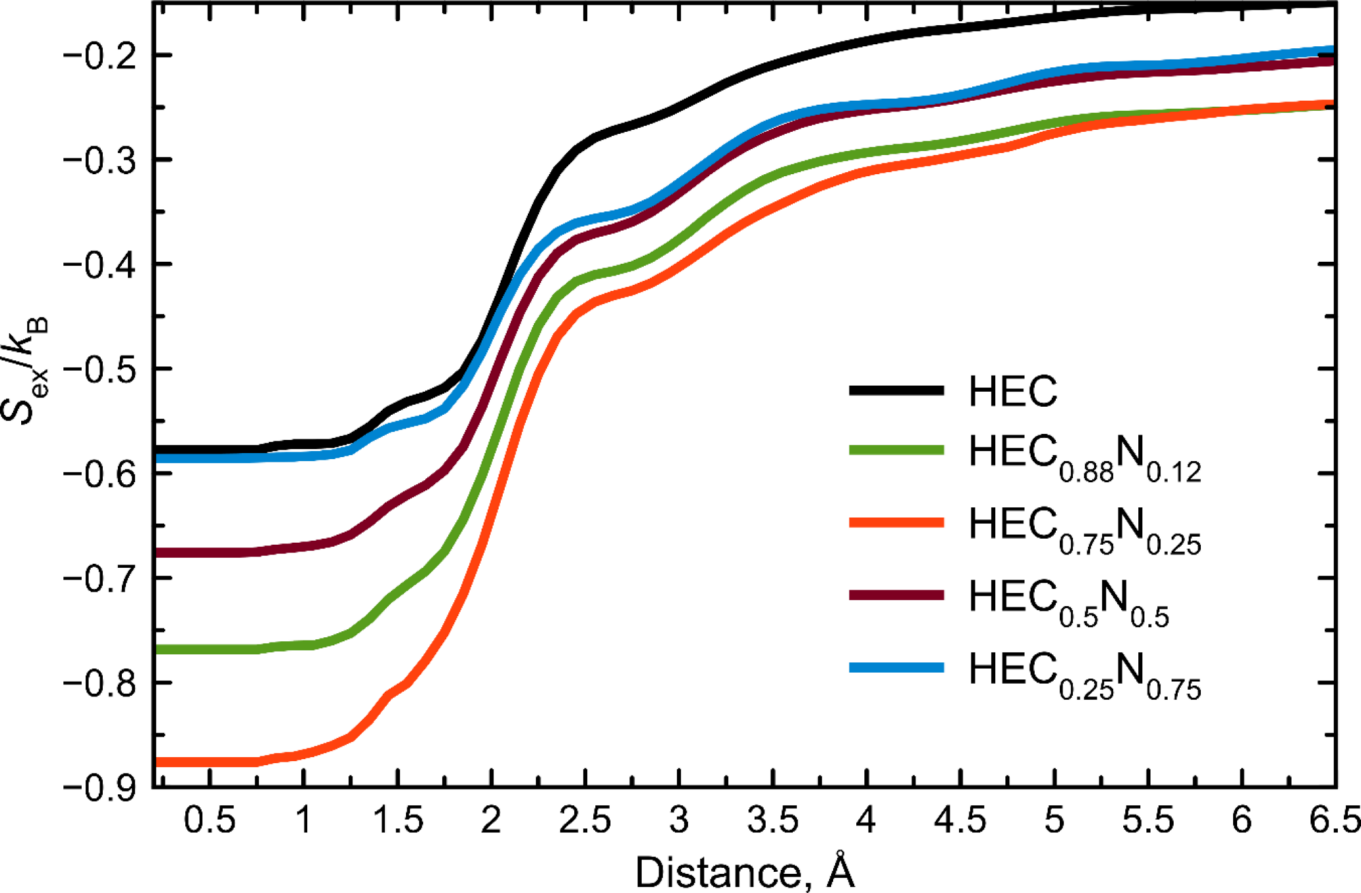
$\:{S}_{ex}/{k}_{B}$ running integral calculated for liquid states of considered carbonitrides with different nitrogen content.

To understand the reason of enhancement of melting temperatures we have calculated the excess entropy for HEC, HEC_0.88_N_0.12_, HEC_0.75_N_0.25_, HEC_0.5_N_0.5_, HEC_0.25_N_0.75_ as shown in Fig. 7. The reduced variety of pair-wise correlations with the addition of N to HEC (forming of HECN) leads to lower entropy of the liquid phase (Fig. 7), which leads to less stability of the liquid phase. On the other hand, formation of the solid solution of carbides and nitrides increases the configurational entropy of the solid HECN phase. This causes the stabilization of the solid HECN phase. Generally, the addition of N should lead to enhancement of the melting point of HECN. As one can see, small amount of nitrogen leads to lowering of entropy of liquid phase and the minimum approaches for HEC_0.75_N_0.25_. Further increase of nitrogen content leads to decreasing the entropy as well as decreasing of melting temperature. Increase of nitrogen content promotes decreasing of stable C-C bonds which are responsible for stability of the liquid phase. Our results correlate with previous investigation of carbonitrides of Hf and Ta^62^, where the maximum melting temperature was achieved for HfC_0.638_N_0.271_, which is close to HEC_0.75_N_0.25_ obtained in our work. We can conclude that efficient mechanism of enhancing melting temperature is addition of nitrogen in the compound, which is promising way for improving stability of functional and constructional materials.

## Conclusions

The melting temperature together with supercooling and superheating temperatures of a number of high-entropy carbonitrides (TiZrTaHfNb)C_x_N_1−x_ with different concentrations of carbon substituted by nitrogen are calculated. The deep learning DeepMD potential is trained on the number of DFT calculations in order to accurately describe both the liquid and solid phases of the considered high-entropy carbonitrides. A new computational procedure is developed and applied to determine the melting hysteresis and melting temperature of HECN using active learning techniques. Heating and cooling curves for HECNs are calculated and carefully analyzed in order to determine the main trends in changes of melting temperatures with respect to nitrogen content. Pair correlation analysis is employed to explain the enhancement of the melting temperature of carbonitrides with small amounts of nitrogen. The obtained data indicates the potential of applications of high-entropy carbonitrides as promising hard and refractive materials for protective coatings of various equipment which operates at extreme conditions.

## Data Availability

The data that support the findings of this study are available from the corresponding authors upon reasonable request.
